# Real-world safety of linaclotide in Chinese patients with irritable bowel syndrome with constipation: a multicenter, single-arm, prospective observational study

**DOI:** 10.1177/17562848251314819

**Published:** 2025-02-05

**Authors:** Yinglian Xiao, Xianmei Meng, Qingfeng Luo, Xiaohua Hou, Jie Jin, Xianfei Zhong, Wei Gong, Xiuling Li, Minhu Chen

**Affiliations:** Department of Gastroenterology and Hepatology, The First Affiliated Hospital of Sun Yat-sen University, Guangzhou, China; Department of Gastroenterology, The Second Affiliated Hospital of Baotou Medical College, Baotou, China; Department of Gastroenterology, Beijing Hospital, Beijing, China; Department of Gastroenterology, Union Hospital, Tongji Medical College, Huazhong University of Science and Technology, Wuhan, China; Department of Gastroenterology, Wenzhou Central Hospital, Wenzhou, China; Department of Gastroenterology, The People’s Hospital of Leshan, Leshan, China; Department of Gastroenterology, Shenzhen Hospital of Southern Medical University, Shenzhen, China; Department of Gastroenterology, Henan Provincial People’s Hospital, Zhengzhou, China; Department of Gastroenterology and Hepatology, The First Affiliated Hospital of Sun Yat-sen University, No. 58 Zhongshan 2nd Road, Yuexiu District, Guangzhou, China

**Keywords:** constipation, guanylate cyclase, irritable bowel syndrome, linaclotide, patient-reported outcomes, safety

## Abstract

**Background:**

Linaclotide, a guanylate cyclase-C agonist, is indicated for irritable bowel syndrome with constipation (IBS-C). However, real-world data on the safety and patient-reported outcomes (PROs) of linaclotide are scarce in Chinese patients with IBS-C.

**Objectives:**

To assess the real-world safety and PROs of linaclotide in the Chinese IBS-C population.

**Design:**

Multicenter, prospective observational study.

**Methods:**

Adults with IBS-C who had taken or planned to take at least one dose of linaclotide 290 μg were enrolled and followed up for 3 months. Face-to-face visits were conducted at baseline (V1), Week 4 ± 7 days (V2), and Week 12 ± 7 days (V3). Primary endpoints included the incidences of adverse events (AEs), AEs by severity, adverse drug reactions (ADRs), serious AEs (SAEs), and AEs leading to treatment interruption, discontinuation, and death. Secondary endpoints included mean treatment satisfaction at V2 and V3, and mean overall Irritable Bowel Syndrome-Quality of Life (IBS-QoL) at V2.

**Results:**

Out of 3000 enrolled patients, 2963 took at least one dose of linaclotide and were analyzed. Overall, 712 patients (24.0%) reported 1095 AEs, which were mostly mild (89.9%). Diarrhea, reported in 297 out of the 2963 patients analyzed (10.0%), was the most common AE. No severe diarrhea was reported. Totally, 319 patients (10.8%) reported ADRs. Forty-six patients (1.6%) reported 50 SAEs and two cases were considered related to linaclotide treatment. Fifty-one (1.7%) and 70 patients (2.4%) interrupted and discontinued treatment due to AEs, respectively. One patient died of hepatic cancer, which was considered unrelated to linaclotide treatment. During the follow-up, the mean (±SD) treatment satisfaction increased numerically and continuously (V1, 2.8 ± 1.3 (*n* = 1721); V2, 3.5 ± 1.1 (*n* = 1705); V3, 3.9 ± 1.0 (*n* = 833)). The mean (±SD) overall IBS-QoL increased numerically from 73.2 ± 16.6 (*n* = 1924) at V1 to 80.2 ± 15.5 (*n* = 1738) at V2.

**Conclusion:**

In the Chinese real-world setting, linaclotide was safe and well tolerated in patients with IBS-C. Numerically, there are trends toward improvement in PROs with linaclotide treatment.

## Introduction

Irritable bowel syndrome (IBS) is a chronic, gut–brain interaction disorder characterized by abdominal pain that is related to changes in bowel habits.^1,2^ The estimated global prevalence of IBS was 9.2% and 3.8% according to Rome III and Rome IV criteria, respectively.^3^ In China, the prevalence of IBS was estimated to be 7.4% (Rome III criteria) and 2.3% (Rome IV criteria) based on an Internet survey, and 3.8% (Rome III criteria) and 1.4% (Rome IV criteria) based on a household survey.^4^

The quality of life of patients with IBS, influenced by factors such as gastrointestinal symptoms and psychiatric conditions, is markedly lower than that in the general population in both physical and mental aspects.^5^ In Chinese patients with IBS, gastrointestinal symptoms such as abdominal pain, discomfort, and bloating are common, with more than half experiencing overlapping abdominal symptoms.^6^ Anxiety and depression, which are common psychiatric conditions that interact bi-directionally with IBS,^7^ are, respectively, reported in 62.1% and 28.9% of patients with IBS in China.^8^ Therefore, therapeutic intervention that can improve the quality of life in patients with IBS is much desired.

IBS consists of four subtypes: IBS with constipation (IBS-C), IBS with diarrhea (IBS-D), IBS with mixed bowel habits (IBS-M), and IBS unclassified (IBS-U).^9–11^ The IBS-C subtype comprises 26.3% of Chinese patients with IBS.^12^ Linaclotide is a guanylate cyclase-C (GC-C) agonist that has been approved to treat IBS-C in the United States, Europe, and China among others. Linaclotide, consisting of 14 amino acids and minimally absorbed by the gut,^13^ activates GC-C on the intestinal epithelium, which promotes the production of cyclic guanosine monophosphate (cGMP) in epithelial cells.^13–16^ The resulting rise in intracellular cGMP concentration increases fluid secretion into the intestinal lumen, which improves intestinal motility and alleviates constipation.^13–16^ Moreover, cGMP can desensitize pain receptors after being transported out of the epithelial cells, thus alleviating abdominal pain.^14–17^

Several North American Phase III trials and a Japanese Phase III trial have demonstrated the efficacy and safety of linaclotide in treating IBS-C.^18–21^ An international Phase III trial that primarily recruited Chinese patients further confirmed that linaclotide treatment was associated with significantly greater improvements in bowel habits and abdominal symptoms (e.g., abdominal pain, discomfort, and bloating) compared to placebo treatment.^22,23^ The trial also reported that diarrhea was the most common adverse event (AE) in the linaclotide group, but discontinuation due to diarrhea was rare.^22,23^ These favorable results led to the approval of linaclotide 290-μg oral capsule by the National Medical Products Administration (NMPA) of China in 2019.

Marketing authorization holders are required by NMPA to further assess the safety of their newly approved drugs in the Chinese population within 5 years of approval. Moreover, there is a lack of large-scale, real-world studies to examine the impact of linaclotide on the quality of life and treatment satisfaction of patients with IBS-C. Thus, in this study, we aim to further examine the safety and patient-reported outcomes (PROs) of linaclotide treatment in Chinese patients with IBS-C.

## Methods

### Study design and participants

This was a multicenter, single-arm, prospective observational study conducted at 30 clinical centers in China (registered with ClinicalTrials.gov, identifier NCT04462900). Eligible patients were enrolled consecutively to minimize selection bias arising from site selection and patient selection. Patients with IBS-C were included if they (a) were at least 18 years old, (b) had taken at least one dose of linaclotide or been prescribed and agreed to start taking linaclotide, (c) were not participants of any interventional study within 3 months before enrollment, and (d) provided written informed consent prior to any study procedures. Patients were excluded if they (a) had contraindications to linaclotide, (b) were unable to comply with the study procedures, or (c) had previously participated in the current study.

The study was reviewed and approved by the Ethics Committee of The First Affiliated Hospital of Sun Yat-sen University (Guangzhou, China; approval number [2019]458-2) as well as the Ethics Committees of all other participating sites. The study was performed in accordance with the Declaration of Helsinki and Good Clinical Practice. All patients provided written informed consent prior to study enrollment. The reporting of this study conforms to the Strengthening the Reporting of Observational Studies in Epidemiology (STROBE) statement.^24^

### Procedures

The initiation and dosing of linaclotide (290-μg oral capsule) were at the physician’s discretion. There was no limitation on concomitant medications during linaclotide treatment. Linaclotide could be discontinued by either the physician or the patient. Patients were followed up for 3 months and face-to-face visits were conducted at baseline (V1), Week 4 ± 7 days (V2), and Week 12 ± 7 days (V3). Patients who discontinued linaclotide during the study period were followed up with at least one phone call within 7 days after discontinuation.

Patients were asked to report AEs during each face-to-face visit and, for those who discontinued linaclotide during the study period, via phone within 7 days after discontinuation. AEs were coded using Medical Dictionary for Regulatory Activities, version 25.1 (MedDRA v25.1) and graded by patients as mild (awareness of sign or symptom, but easily tolerated), moderate (discomfort sufficient to cause interference with normal activities) or severe (incapacitating, with inability to perform normal activities).

Patients completed the Irritable Bowel Syndrome-Quality of Life (IBS-QoL) questionnaire at V1 and V2. IBS-QoL consists of eight subdomains: dysphoria, interference with activity, body image, health worry, food avoidance, social reaction, sexuality, and relationship. An overall IBS-QoL score between 0 and 100 was calculated based on all subdomain scores, with 100 indicating the highest quality of life. Patients also reported treatment satisfaction (on an ordinal scale of 1–5, with 5 being the most satisfied) at V1, V2, and V3.

### Outcomes

The primary endpoints were numbers and proportions of patients who reported AEs, AEs by severity, ADRs, serious AEs (SAEs), and AEs leading to linaclotide interruption, discontinuation, and death. The relationship between AEs and linaclotide treatment was assessed by investigators. The secondary endpoints were PROs including the mean overall IBS-QoL score at V2 and the mean treatment satisfaction score at V2 and V3.

Prespecified subgroup analysis of PROs was conducted in patients according to treatment history. Patients were divided into the new treatment group, consisting of those who did not receive linaclotide within 1 month before enrollment, and the used treatment group, consisting of those who received linaclotide within 1 month before enrollment. All analyses for the secondary endpoints were repeated for both subgroups.

Moreover, for the new treatment group only, the mean change from baseline in overall IBS-QoL scores at V2, mean change from baseline in treatment satisfaction at V2 and V3, and the number and proportion of patients with clinically meaningful improvement in overall IBS-QoL score (improvement ⩾ 14) at V2,^25,26^ were further assessed.

### Statistical analysis

The sample size of 3000 was determined to meet NMPA’s requirement for post-authorization drug-intensive monitoring programs. Safety was evaluated in the safety population, which consisted of all patients who received at least one dose of linaclotide, whereas PROs were assessed in the efficacy population, which consisted of all patients who received at least one dose of linaclotide and had at least one non-missing baseline and one post-baseline PRO parameter. Missing data were not imputed (missing = excluded). All results were descriptively summarized. The 95% confidence intervals (CIs) for the incidences of AEs and ADRs were calculated using the Clopper–Pearson method.

## Results

### Study population

**Figure 1.**
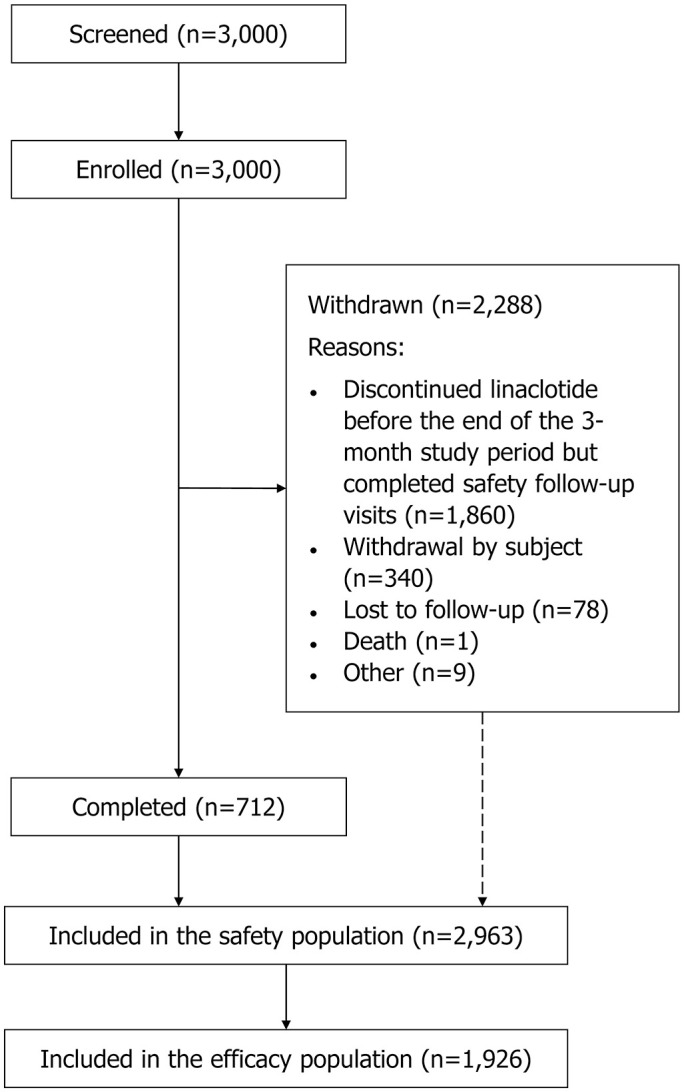
Patient flow diagram.

From 18 September 2020 to 31 October 2022, 3000 patients with IBS-C from 30 centers/tertiary hospitals in China were enrolled. By the data cutoff, 712 (23.7%) patients completed the study and 2963 patients received at least one dose of linaclotide and were thus included in the safety population (Figure 1). A total of 1926 patients were included in the efficacy population (Figure 1), with 1482 (76.9%) in the new treatment group and 444 (23.1%) in the used treatment group. In the safety population, the mean age (±standard deviation [SD]) was 49.8 ± 16.5 years, and 2131 (71.9%) patients were female (Table 1).

**Table 1.** Patient demographics and baseline characteristics.

Variable	Patients, *n*	Value
Mean age ± SD, years	2963	49.8 ± 16.5
Female, *n* (%)	2963	2131 (71.9)
Mean height ± SD, cm	2951	163.7 ± 7.3
Mean weight ± SD, kg	2951	59.9 ± 10.2
Mean BMI ± SD, kg/m^2^	2951	22.3 ± 3.1
Median time since IBS-C diagnosis (range), years	654	0.0 (0.0, 26.2)
Mean overall IBS-QoL score ± SD	1924	73.2 ± 16.6
Mean dysphoria subdomain score ± SD		72.8 ± 19.6
Mean interference with activity subdomain score ± SD		77.4 ± 18.5
Mean body image subdomain score ± SD		84.5 ± 17.9
Mean health worry subdomain score ± SD		60.6 ± 22.6
Mean food avoidance subdomain score ± SD		69.0 ± 22.2
Mean social reaction subdomain score ± SD		81.5 ± 18.2
Mean sexual subdomain score ± SD		89.7 ± 18.5
Mean relationship subdomain score ± SD		85.8 ± 18.1
Mean treatment satisfaction score ± SD	1721	2.8 ± 1.3

BMI, body mass index; IBS-C, irritable bowel syndrome with constipation; IBS-QoL, irritable bowel syndrome-quality of life; SD, standard deviation.

### Safety

Within the safety population, AEs were reported in 712 (24.0%, 95% CI (22.5%, 25.6%)) patients (Table 2). Most patients (*n* = 679, 95.4%) reported mild or moderate AEs, while 33 (4.6%) patients reported severe AEs (Table 2). The most common AE was diarrhea, which was reported in 297 (10.0%) patients. All cases of diarrhea were mild or moderate in severity (Table 3). Each of all other AEs was reported by fewer than 1% of patients. ADRs were reported by 319 (10.8%, 95% CI (9.7%, 11.9%)) patients (Table 2). Diarrhea was the most common ADR, reported by 289 (9.8%) patients (Table 3).

**Table 2.** Summary of adverse events.

AEs	Patients (*N* = 2963) *n* (%, (95% CI))	Number of events
Any AEs	712 (24.0, (22.5, 25.6))	1095
Mild	615 (20.8, (19.3, 22.3))	984
Moderate	64 (2.2, (1.7, 2.8))	75
Severe	33 (1.1, (0.8, 1.6))	36
ADRs	319 (10.8, (9.7, 11.9))	361
SAEs	46 (1.6, (1.1, 2.1))	50
AEs leading to treatment interruption	51 (1.7, (1.3, 2.3))	56
AEs leading to discontinuation	70 (2.4, (1.9, 3.0))	74
AEs leading to death	1 (0, (0, 0.2))	1

ADR, adverse drug reaction; AE, adverse event; SAE, serious adverse event.

**Table 3.** Summary of diarrhea adverse events.

AEs	Patients (*N* = 2963), *n* (%)	Number of events
Diarrhea as AE	297 (10.0)	323
Mild	281 (9.5)	307
Moderate	16 (0.5)	16
Severe	0 (0)	0
Diarrhea as ADR	289 (9.8)	315
Diarrhea as SAE	0 (0)	0
Diarrhea leading to discontinuation	54 (1.8)	54

ADR, adverse drug reaction; AE, adverse event; SAE, serious adverse event.

AEs led to interruption of linaclotide in 51 (1.7%, 95% CI (1.3%, 2.3%)) patients and discontinuation in 70 (2.4%, 95% CI (1.9%, 3.0%)) patients (Table 2). The most common cause for discontinuation was diarrhea (77.1% (54/70)), followed by abdominal pain (10.0% (7/70)) and abdominal distension (4.3% (3/70)).

SAEs were infrequent and reported in 46 (1.6%) patients (Tables 2 and 4). Most SAEs were assessed to be unrelated to linaclotide treatment, except for one case of moderate hemorrhoid and one case of spontaneous abortion, which were considered by investigators to be likely related to linaclotide treatment. One patient died of AE (hepatic cancer) during the follow-up period, which was assessed to be unrelated to linaclotide treatment.

**Table 4.** Summary of serious adverse events.

SAEs	Patients (*N* = 2963) *n* (%)	Number of events
All SAEs	46 (1.6)	50
Gastrointestinal disorders		
Large intestine polyp	11 (0.4)	11
Constipation	3 (0.1)	3
Hemorrhoids	3 (0.1)	3
Gastric polyps	2 (0.1)	2
Abdominal adhesions	1 (0.0)	1
Chronic gastritis	1 (0.0)	1
Duodenal ulcer	1 (0.0)	1
Gastroesophageal reflux disease	1 (0.0)	1
Ileus	1 (0.0)	1
Proctalgia	1 (0.0)	1
Other categories of SAEs		
Angina pectoris	2 (0.1)	2
Cholelithiasis	2 (0.1)	2
Abortion spontaneous	1 (0.0)	1
Cardiac failure	1 (0.0)	1
Cerebral infarction	1 (0.0)	1
Cholangitis infective	1 (0.0)	1
Cholecystitis	1 (0.0)	1
Craniocerebral injury	1 (0.0)	1
Depression	1 (0.0)	1
Diabetes mellitus	1 (0.0)	1
Ectopic pregnancy	1 (0.0)	1
Eye hemorrhage	1 (0.0)	1
Glomerulonephritis	1 (0.0)	1
Head discomfort	1 (0.0)	1
Hepatic cancer	1 (0.0)	1
Hyperparathyroidism	1 (0.0)	1
Hypokalemia	1 (0.0)	1
Lower limb fracture	1 (0.0)	1
Muscular weakness	1 (0.0)	1
Neck pain	1 (0.0)	1
Respiratory tract infection	1 (0.0)	1
Squamous cell carcinoma of the cervix	1 (0.0)	1
Wolff-Parkinson-White syndrome	1 (0.0)	1

SAE, serious adverse event.

### Patient-reported outcomes

**Figure 2.**
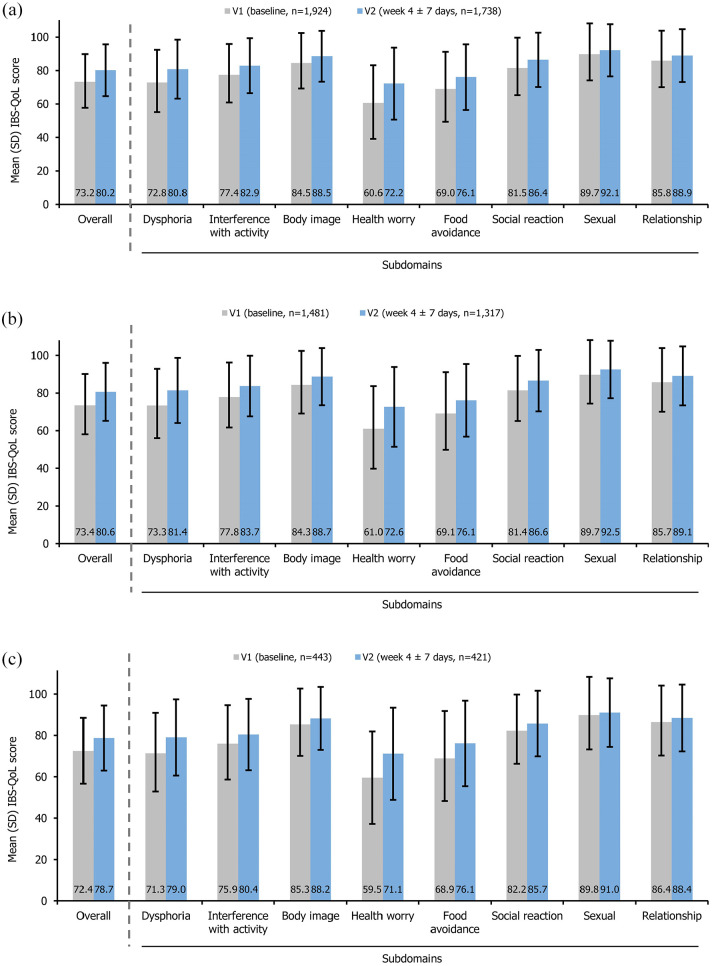
Mean (SD) overall and subdomain IBS-QoL scores in (a) the efficacy population, (b) the new treatment group, and (c) the used treatment group. IBS-QoL, irritable bowel syndrome-quality of life; SD, standard deviation.

Based on patients with available IBS-QoL scores at study visits, the mean (±SD) overall IBS-QoL score increased numerically from 73.2 ± 16.6 (*n* = 1924) at V1 to 80.2 ± 15.5 (*n* = 1738) at V2, with the mean IBS-QoL scores increasing for all subdomains (Figure 2(a)). The new treatment group and the used treatment group demonstrated a similar extent of increase in overall and sub-domain IBS-QoL scores (Figure 2(b) and (c)). In the new treatment group, the mean (±SD) change from baseline in overall IBS-QoL score was 6.9 ± 13.7 (*n* = 1317) at V2. Clinically meaningful improvement in overall IBS-QoL score, defined as ⩾14 improvement in overall IBS-QoL score, was observed in 22.5% (334/1482) of patients.

**Figure 3.**
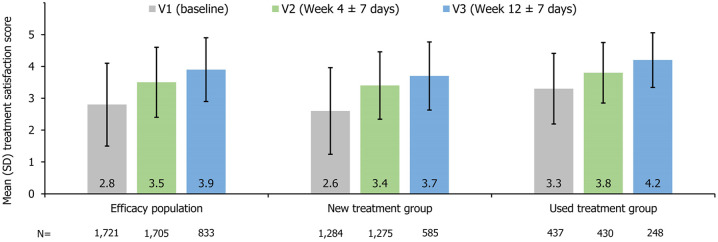
Mean (SD) treatment satisfaction in the efficacy population, the new treatment group, and the used treatment group. SD, standard deviation.

In patients with available data on treatment satisfaction, the mean (±SD) overall treatment satisfaction score increased numerically from 2.8 ± 1.3 (*n* = 1721) at V1 to 3.5 ± 1.1 (*n* = 1705) at V2 and further increased to 3.9 ± 1.0 (*n* = 833) at V3 (Figure 3). The proportion of patients who reported “quite satisfied” (score = 4) or “very satisfied” (score = 5) increased from 29.8% (512/1721) at baseline to 48.8% (832/1705) at V2 and 62.3% (519/833) at V3, based on patients whose treatment satisfaction data were available at the respective visits. Both the new treatment group and the used treatment group demonstrated a numerically increasing trend in treatment satisfaction (Figure 3). In the new treatment group, the mean (±SD) changes from baseline in treatment satisfaction were 0.7 ± 1.3 (*n* = 1275) and 0.8 ± 1.6 (*n* = 585) at V2 and V3, respectively, in patients with data on treatment satisfaction.

## Discussions

To our knowledge, this is the largest real-world study to date that assessed the safety and PROs associated with linaclotide in Chinese patients with IBS-C. In the present study, the reported occurrence of AEs (24.0%) was low with no new safety signal identified. The two SAEs (one moderate hemorrhoid and one spontaneous abortion) that are currently not listed in the prescribing information^27^ were considered by investigators to be likely related to linaclotide treatment. However, the two SAEs were not considered as new safety signals based on the following reasons: on the one hand, linaclotide is minimally absorbed^13^ and, therefore, unlikely to cause systemic side effects; on the other hand, information on the two SAEs was limited (e.g., detailed etiologic workup was unknown). Of patients who reported at least one AE in this study, the majority (95.4%) reported mild to moderate AEs. The rates of treatment disruption and discontinuation due to AEs (1.7% and 2.4%, respectively) in the safety population were low, demonstrating the high tolerability of linaclotide in real-world settings. Moreover, the mean overall IBS-QoL and treatment satisfaction scores both improved during the follow-up, indicating that linaclotide exerted a positive impact on PROs.

This study further confirmed the safety and tolerability of linaclotide in Chinese patients with IBS-C. Compared with previous publications, the 12-week reported rate of AEs was lower than those reported by the international Phase III trial, the Japanese Phase III trial, and a North American trial (24.0% vs 30.3%–56.2%).^20,21,23^ Such observation could be attributed to the lower proportion of patients who completed the 12-week treatment with linaclotide in this real-world study than those in previous trials with stricter treatment protocols (23.7% vs 76.8%–92.1%).^20,21,23^

Consistent with previous studies, diarrhea was the most reported AE.^18–23,28–31^ The proportion of patients reporting diarrhea in this study (10.0%) was similar to those in a previous Chinese real-world study conducted among patients with IBS-C and a Chinese sub-cohort analysis of the aforementioned international Phase III trial (11.3% and 8.3%, respectively),^22,32^ but much lower than that in the previous North American trial (19.5%).^20^ The low rate of treatment discontinuation due to AEs in this study was also consistent with the aforementioned Chinese sub-cohort analysis (2.4% vs 1.8%).^22^ In general, in the real-world setting of our study, linaclotide was safe, and well-tolerated, with a safety profile consistent with that reported in clinical trials.

The present study has also added more weight to existing evidence that linaclotide can improve PROs in patients with IBS-C. In this study, the IBS-QoL questionnaire, a validated instrument for the Chinese population,^33^ was used to reliably quantify the quality of life in patients with IBS-C. Earlier Phase III trials from North America and a real-world study from the UK have shown that IBS-QoL improved after 12 weeks of treatment with linaclotide in patients with IBS-C,^19,31^ but the short-term benefit of linaclotide on IBS-QoL remained to be explored. In our study, based on patients with available IBS-QoL scores at study visits, the mean overall and subdomain IBS-QoL scores improved numerically as early as 4 weeks after linaclotide treatment, which was consistent with the previous Japanese Phase III trial and a small-scale Chinese real-world study.^21,32^ Furthermore, numerical increases in overall and subdomain IBS-QoL scores were demonstrated in both the used and new treatment groups, suggesting that continued treatment with linaclotide could consistently improve quality of life. Of note, a substantial proportion of patients (22.5%) in the new treatment group experienced clinically meaningful improvement in overall IBS-QoL score (improvement ⩾ 14) only 4 weeks after treatment initiation. As IBS-QoL score tends to increase further with continued treatment as shown in the Japanese Phase III trial and the British real-world study,^21,31^ more patients might have experienced clinically meaningful improvement in IBS-QoL at the end of follow-up in our study.

Treatment satisfaction is another important PRO parameter that was evaluated in the present study. Based on patients with available treatment satisfaction data at study visits, it was observed that treatment satisfaction increased numerically from baseline at week 4 and further increased numerically at week 12. This finding is consistent with the results of the aforementioned Chinese sub-cohort analysis, where the mean treatment satisfaction demonstrated an increasing trend from baseline to week 12.^22^ At week 12, 62.3% of patients with available treatment satisfaction data for this timepoint in our study were quite or very satisfied with linaclotide treatment, which was somewhat higher than that reported in the previous North American trial (52%).^20^ Therefore, our study further reinforces existing evidence, highlighting the positive impact of linaclotide on improving treatment satisfaction in patients with IBS-C.

The results of this study should be interpreted in light of its potential limitations. First, the study was non-interventional with no comparator arms involved. Second, a high proportion of patients withdrew from the study before the end of the 3-month follow-up period. This resulted in shorter overall follow-up than if most patients completed the 3-month linaclotide treatment, which could further cause the underestimation of the occurrences of AEs. Selection bias might have also arisen due to the large proportion of withdrawal, particularly for the PRO results, as patients who discontinued treatment before week 12 could be dissatisfied with treatment. The extent of selection bias could not be assessed given the current data. Third, underreporting of AEs might have occurred in this study as AEs were reported based on patients’ memories, which might lead to recall bias. A further limitation is that the 3-month follow-up period used in this study is relatively short, making it difficult to capture potential chronic or delayed adverse reactions. Yet, considering that linaclotide is minimally absorbed,^13^ the probability of developing such adverse reactions might be low. Moreover, IBS-QoL was only assessed at week 4, and no further assessment was arranged at the end of the follow-up so that the durability of the improvement in IBS-QoL scores could not be demonstrated. Nevertheless, as discussed above, previous studies have shown that the IBS-QoL score tends to increase further as treatment continues.^21,31^ Lastly, only descriptive analyses were planned for and conducted in this study. Consequently, the predictors of AEs and improvements in PROs, and the statistical significance of the improvements in PRO parameters were not assessed, as they are beyond the scope of the study protocol. Conducting post hoc analyses on these aspects would have required obtaining approval from the study sites to draft a new statistical analysis plan for post hoc analyses, perform the analyses, and update the study summary report. However, as all 30 participating study sites were closed upon study completion, it was no longer feasible to obtain approval for additional post hoc analyses from the sites or their respective ethics review committees. Nevertheless, we consider it of limited value to conduct further statistical analysis based on the available data. Due to the presence of potential confounders and biases (such as selection bias mentioned above) in this observational study, it would be difficult to directly attribute any observed significant differences to specific factors. A future study using designs that better control for bias is warranted to address the abovementioned research questions.

## Conclusion

In the real-world clinical setting, linaclotide was safe and well tolerated in adult patients with IBS-C in China. AEs occurred at a low incidence and were mostly mild, with diarrhea as the most reported AE. Numerically, there are trends toward improvement in treatment satisfaction and quality of life in the Chinese IBS-C population treated with linaclotide. The consistency of the results of our study with those of previous trials further supports linaclotide as a valuable treatment option for patients with IBS-C.

## Declarations

**Ethics approval and consent to participate:** The study was reviewed and approved by the Ethics Committee of The First Affiliated Hospital of Sun Yat-sen University (Guangzhou, China; approval number [2019]458-2) as well as the Ethics Committees of all other participating sites. All patients provided written informed consent prior to study enrollment.

**Consent for publication:** Not applicable.

**Availability of data and materials:** The datasets used and/or analyzed during the current study are available from the corresponding author upon reasonable request.
